# General data protection regulation: an algorithmic proposal for forensic photography

**DOI:** 10.1007/s00414-025-03640-w

**Published:** 2026-02-04

**Authors:** Mariana Cura, Ricardo Loureiro, Pedro Marcelino, Vanessa Rodrigues, José Paulo Andrade

**Affiliations:** 1 Instituto Nacional de Medicina Legal e Ciências Forenses – Delegação do Sul, Rua Manuel Bento de Sousa 3, Lisboa, 1169-201 Portugal; 2 Independent Researcher, Largo do Souto 46, Custoias, 4460-670 Portugal; 3 Cintesis@Rise Unit of Anatomy, Department of Biomedicine, Faculty of Medicine, University of Porto, Porto, 4200-450 Portugal

**Keywords:** Forensic photography, Photography, Smartphones, Privacy, Informed consent, Data security

## Abstract

Since 2018, the General Data Protection Regulation (GDPR) has regulated personal data within the scope of the European Union. With the exponential technological advancements in mobile photography, it is crucial to expose forensic professionals to this body of law to maintain good practices for fieldwork and scientific research in this field. GDPR, as far as its application in forensic photography, can be broken down into four pillars: informed consent of the subject, acceptable image capture practices (data), data storage and security at rest, and data transfers and security in transit. All these pillars have different approaches currently in use by forensic professionals. However, only some of them are permitted under the law. We present the appropriate ways to proceed with smartphone photography while remaining in compliance and maintaining the ability to share data critical to fieldwork and scientific research. In addition, some of the common pitfalls are described. An algorithm is proposed to facilitate compliance with European regulations relating to personal data, as applied to mobile forensic photography. The same flow chart can be used in other countries with different regulations concerning health data, privacy, and security issues.

## Highlights

- General Data Protection Regulation (GDPR) controls data including medical photography.
- GDPR mandates careful attention to data collection, storage, and transfer.
- Mobile photography demands that forensic professionals are versed in GDPR.
- Standardizing the image collection is necessary to maintain quality and consistency.
- An algorithm is proposed to obtain legal compliance when using smartphones.

## Introduction

Innovations in the field of miniaturized image sensors have reached a point where companies advertise movies that were shot entirely with a mobile phone [1]. In recent years, these technological advancements have given an ever-broader range of people the ability to take sharp, clear photographs at a moment’s notice, all with a device most carry daily and without the need or training for bulky and expensive equipment that was previously needed to achieve apparently similar results [2].

Medical photography is essential for documenting, examining, and proving clinical findings, especially in forensic science, as these photographs can be used as evidence in a criminal investigation and civil law [3]. In a hospital setting, smartphones are becoming the most common tool for capturing a medical photograph [4, 5], to document patients clinical progress and ask for second opinions [4–6], as images give more easily comprehensible evidence than a written assessment and have the advantage of being quickly interpreted when shared with others [7]. Newer generations of doctors also tend to use their smartphones to obtain images in comparison to senior ones’ [2, 5], as the devices are readily available and can obtain a photograph anytime, anywhere [8]. Several reports demonstrate that using smartphones in clinical settings is widely accepted by patients, particularly if the institution owns the devices [7, 9–12]. Traditional digital cameras are more readily found in the forensic field, where professionals use them to document lesions, such as marks, bruises, and entry and exit wounds, in both deceased and living subjects [7, 13]. Medical photography proves itself especially important in cases of added complexity, where it has been used to better illustrate the relevant evidence and can impact the course of a legal case [7, 13].

For the purposes of this article, we define a smartphone as a mobile phone performing most of the functions of a computer and being capable of running various software applications known as apps [2]. Medical photography is the capture of an individual’s appearance in the context of clinical or forensic work, including hospitalization, clinical or forensic appointment settings, surgical procedures, pathology, and autopsies, amongst others.

Extensive literature exists on the correct procedure for taking photographs using digital cameras in the clinical and forensic field, and sometimes, professional forensic photographers are available [14]. Unfortunately, while medical professionals in a hospital setting have some, albeit limited, literature regarding the correct use of smartphone photography [15–17], the forensic field has not. Medical professionals in the forensic field need to know how to capture photographs with mobile devices if they cannot use their institutional equipment for unforeseen reasons, such as battery drainage and damage to the equipment, or are not provided with any such equipment in the first place.

However, paying attention to the General Data Protection Regulation (GDPR) is crucial when photographing with a personal device such as a smartphone. GDPR is a comprehensive data protection law that came into effect in the European Union (EU) on May 25, 2018 [18]. As such, personal data and its collection, including photography of the human body for medical purposes, has been regulated within the EU [19]. Unfortunately, most health organizations or institutions are not able to accompany the evolution of the technology, mainly in the critical issues related to acquisition, management, storage, and access to clinical and forensic images [7]. It also appears that the standards for the acquisition of images using smartphones are not regulated or structured [7], the quality obtained is inconsistent, and the training is minimal or non-existent [7].

Patient privacy rules are becoming more complex, and the legal consequences of violations are escalating. Therefore, exposing forensic professionals to this body of law is now crucial to maintaining good practices for both fieldwork and scientific research.

## GDPR made simple

Depending on the country’s location, different regulations and laws may be at play because of the sensitive nature of personal and health data. The exact terminology of these laws varies among countries and judicial systems, but the concepts remain the same [20]. In countries where the GDPR is at play, the law demands consideration of the underlying principles of security, privacy, consent, and fair data collection [21]. As far as the application of GDPR in patients and forensic photography goes, independently of the current setting, be it clinical or forensic; we have, for the purpose of accessibility and ease of reference, broken it down into four main pillars:

1. Informed consent
2. Acceptable image capture practices (data collection)
3. Data storage and security at rest
4. Data transfers and security in transit

Though GDPR refers to live subjects, it “does not apply to the personal data of deceased persons. Member States may provide for rules regarding the processing of personal data of deceased persons” (Recital 27 [19]). However, the authors feel it is important to exercise the same level of care for the data gathering, processing, storage, transfer, and security of forensic information regarding the deceased, given the sensitive nature of the cases at hand, and to respect the living memories of those who knew the person.

 For the purposes of this article and considering GDPR, here are some definitions:

- **Data processing** refers to “any operation or set of operations which is performed on personal data or sets of personal data, whether or not by automated means, such as collection, recording, organisation, structuring, storage, adaptation or alteration, retrieval, consultation, use, disclosure by transmission, dissemination or otherwise making available, alignment or combination, restriction, erasure or destruction” (Article 4 [19])
- **Personal data** refers to “any information relating to an identified or identifiable natural person (‘data subject’)” (Article 4 [19])

###  Informed consent

 Institutional policies and procedures should be implemented to ensure that consent is obtained, and access control is adequate [7]. As stated in Recital 32 of GDPR [19]:

“Consent should be given by a clear affirmative act establishing a freely given, specific, informed, and unambiguous indication of the data subject’s agreement to the processing of personal data relating to him or her, such as by a written statement, including by electronic means, or an oral statement.”

“Consent should cover all processing activities carried out for the same purpose or purposes. When the processing has multiple purposes, consent should be given for all of them.”

“Silence, pre-ticked boxes or inactivity should not, therefore, constitute consent” (when applied to living subjects).

Oral or written consent must be obtained [8, 19]. Before consent can be given, the medical professional must explain:

- What is the purpose of capture
- Who will be responsible for data security
- Where will the data be stored, temporarily and permanently
- How the images are transferred
- Whom it may be shared with
- How to withdraw or withhold consent, which is possible at any time during data collection
- Consent for journal submissions should be obtained at the time of consultation [22].

Depending on how the consent is obtained:

- Oral consent – register that consent was given after providing relevant information to the data subject in the final report
- Written consent – file with final report

If the subject is alive but is unable to give informed consent (such as in cases of disability, under-age, unconsciousness, etc.), the persons’ legal representative must give consent. In exceptional cases where time is of the essence to capture the findings, and the time for acquisition of consent exceeds the time to take the photograph, consent can be obtained after capture, with the understanding that they must be deleted if consent is not given.<div class="NodiCopyInline">If the subject is alive but is unable to give informed consent (such as in cases of disability, under-age, unconsciousness, etc.), the persons’ legal representative must give consent. In exceptional cases where time is of the essence to capture the findings, and the time for acquisition of consent exceeds the time to take the photograph, consent can be obtained after capture, with the understanding that they must be deleted if consent is not given.

However, the requirement of consent is not always present or expected, such as when collecting evidence as mandated by a court of law [23].

###  Acceptable image capture practices (data collection) using a mobile device

A great medical photograph can conceal a patient’s identity while accurately capturing a clinical finding, showing location, size, colour, texture, and depth [16]. Its quality is critical for clinical value, and attention should be paid to accurate, consistent, sharp, and high-quality images [8]. Smartphone photographs quality is usually acceptable, and some smartphones deliver very good images and the ability to obtain RAW files [24]. The problem, however, is the easy and rather casual nature of smartphone image acquisition [7], and standardization and consistency are critical to the photographic process when comparing, for example, the evolution of a lesion over time [7], even more so when using a smartphone [2].

If possible, remove identifiers from the frame. An identifier is defined as any feature of a photograph that can be related to a particular patient. Patient armbands with the name, tattoos or unique birthmarks, full facial portraits, and imaging containing the patients’ name are examples of identifiers [20]. Also, when naming files containing clinical images, do not include patients’ names and record numbers [22]. If present in the frame, the image is protected health information, and extra care must be taken to store it securely [20]. Regrettably, security breaches are frequently reported, and clinical images are vulnerable to cyberattacks [22]. For instance, in 2020, a plastic surgery practice in the United Kingdom experienced a breach in which nearly one terabyte of patient image data was compromised, and a ransom demand was issued [22].

Clinicians can reduce inaccurate interpretation of findings and take quality pictures by following these simple parameters of medical photography [15]:

- Lighting.
- Focus and distortion.
- Location and severity.
- Colour and background.
- Perspective.
- File quality.

**Fig. 1:**
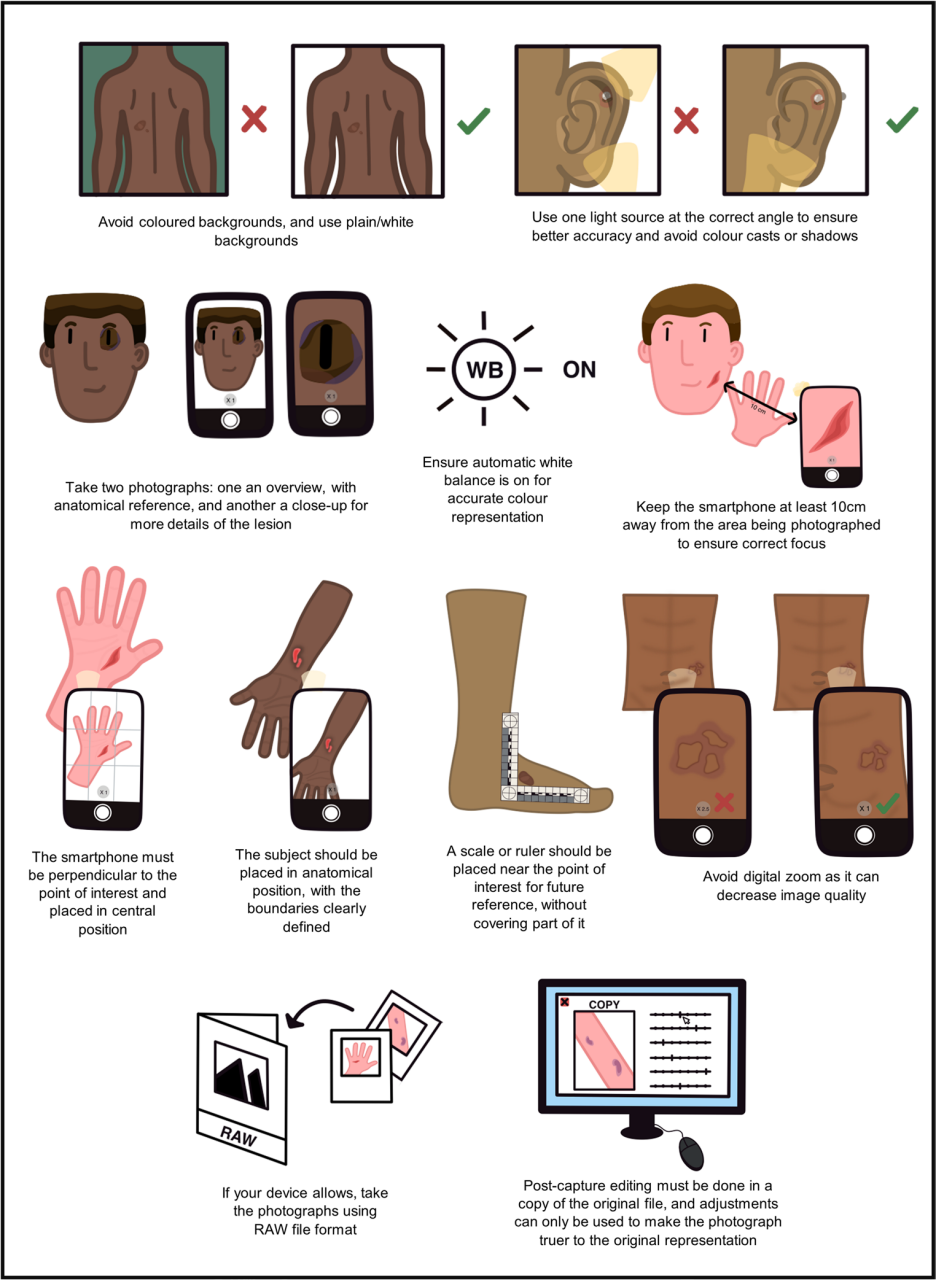
Summary of acceptable image capture practices

Figure 1 Summarises the main take-home messages for acceptable image capture practices, which are described in more detail below.

To apply some of the principles noted, we recommend the use of a camera app other than the default one. While this may not be true for all cases, most phones will come available with an app which will not expose the full range of settings and capabilities of the device, all while applying liberal amounts of tweaks, optimizations, and even artificial intelligence (AI) generation to the end image.

To avoid recommending specific apps, as these may vary over time, we suggest searching for “RAW Camera app” in your search engine. The time spent searching for the ideal app, which bares the most authentic results with a given device, is a one-time commitment with the greatest impact on image capture.

 When searching for the ideal app, look out for, and when possible, test the following criteria for their accuracy between what you see directly, and through the final image:

- Skin tones
- Skin smoothing
- Consistency of contrasting tones

**Lighting** [2, 15] - Aperture, shutter speed, and ISO must be balanced for a correct exposure. Without user interference, most mobile devices will automatically reconfigure these settings. So, if you wish to increase image clarity, increase the quantity of light. This will often trigger the mobile device to decrease the ISO, which leads to the desired effect. The angle of the light source also affects accuracy. A light source or smartphone flash providing good illumination of the subject is important to obtain a sharp image with low ISO, small aperture, and absence of motion blur due to adequate shutter speed. 

 **Focus and distortion** [15, 25, 26] - Focal length can differ throughout devices, and so does the minimum focus distance, with most devices having a minimum distance of 10 cm and some having macro capabilities, allowing the user to get closer to the subject, and often providing greater detail. Distortion of features (particularly facial) occurs with a short distance between the subject and the camera due to the smartphone’s single focal length and can introduce inconsistency. To ensure correct focus and avoid distortion, avoid digital zoom, which can always be achieved by cropping the imager at a later data, and always leads to data loss and decreased image quality; and keep a distance of at least a hands’ width from the subject. Because of the high-resolution capabilities of modern devices, sufficient detail is retained for posterior reference and amplification. Use the zoom only if the smartphone has optical zoom capabilities, and only up to the point before it starts using digital.

 **Location and severity** [2, 15] - Take at least two photographs: one showing an overview of the lesion, with anatomical reference points, and a close-up image showing more detail of the lesion.

**Colour and background** [15, 27, 28] **-** It is essential to reduce inaccurate colour representation, and most users ignore this critical issue. An adequate background should be uniform in colour. Most smartphone cameras have an automatic white balance setting, but they can still present problematic colour errors. In addition, smartphones software utilizes artificial intelligence (AI) and machine-learning technology, presents bias against dark skin tones, and favours lighter skin tones. Using high-end smartphones and adequate apps, ensure that the automatic white balance setting is on, use a plain white background, and use a single source of light (flash or daylight, to avoid colour cast). Avoid coloured backgrounds, clothing with bright colours in the frame, and combining different light sources. In the case of coloured lesions, the same device must be used to capture the lesion. To have reproducibility over time, the same device and settings should be used, the image ideally should be observed on the same calibrated monitor, and if a printer is necessary, the same printer and printing settings should be used.

 **Perspective** [2, 15] - The subject should be placed in the anatomical position, and the smartphone camera must be perpendicular to the point of interest and placed in a central position, with the boundaries clearly defined. A scale or ruler (preferably a photomacrography scale) should be near the point of interest and not cover part of it.

**File quality** [15] - If your device allows it, capture and store all the photographs in RAW file format. This capability is generally present in high-end smartphones. RAW files preserve the most detail, giving files the highest possible image quality, the widest tonal range, and minimal processing without altering or distorting their content. It is possible to change in post-processing, including white balance correction, and it is a lossless format, allowing savings with no loss of quality. Whenever possible, prefer to store the original data in this format. The disadvantage is the necessity to use apps to maximize the quality of the final image, and the size of the files is bigger, occupying more disk space. The most common file format is JPEG. The advantage of JPEG is that practically all computer software and online sites support it. Although universal, it is prone to loss of quality after saving one image several times, creating artifacts, and correcting the white balance is challenging. If possible, the highest resolution JPEG should be chosen. Convert to TIFF or PNG to edit the image as they preserve most of the data. JPG can be used only after the edition and for published works.

Post-capture editing of clinical and forensic photographs using programs such as Lightroom^®^ and Photoshop^®^ can only be used for adjustments that will make the photograph truer to the original, such as colour correction, contrast, brightness, sharpness, and saturation [27]. Some of these changes can be made on the smartphone [8]. These alterations should be made on a copy of the original file [27]. Making gross changes, such as enhancing, deleting, or obscuring specific features, is not permitted [27].

It is also worth noting that most devices will capture the location where a photograph was taken and store it within the photograph’s metadata. Be mindful of this feature when sharing the files.

If applied, the local chain of custody procedures should be followed regarding the medical photographs [7].

It is important to stress that the line between digital cameras and smartphones is increasingly fine as the flagship brands present more sophisticated camera arrays every year. At present, for medical and forensic use, digital cameras with interchangeable lenses that are not prone to distortions present significant advantages in versatility and quality due to the sensor size and resolution and are still considered the gold standard [29]. However, some studies show that the quality of images obtained with smartphones has reached a similar quality to those obtained with digital cameras, allowing clinical decisions [30, 31].

The main advantages of smartphones are convenience and connectivity with acceptable quality if the capture practices shown in Fig. 1 are followed.

These and other main advantages and disadvantages of using a mobile device for medical photography [2] are summarized in Table 1..

**Table 1.** Summary of the main advantages and disadvantages of the use of a mobile device in medical photography

Advantages	Disadvantages
Image transfer can be easy and quick	Auto-sync with cloud storage
Good image resolution	Digital zoom decreases photo precision
User-friendly and intuitive device	Standardization is more difficult with a smartphone camera
Time-saving device	Mobile devices have smaller sensors; the size of the pixels is smaller
Layers of authentication (password, fingerprint, two-factor authentication)	Macro mode in a mobile device does not match one in a digital camera; very prone to lens distortion
Cost-friendly device	Tendency for over exposure
Information can be deleted remotely if misplaced or stolen	Possibility of alteration of skin tone representations due to artificial intelligence algorithms

Based mostly on reference [2].

###  Data storage and security at rest

Professionals and institutions are sometimes unaware of the importance of data storage and security at rest, and its importance due to the sensitive nature of health and forensic data [7, 32]. Note that in the case of forensic photographs, there is a potential classification of this type of data as evidence, and legal confidentiality obligations [22].

Data storage applies to all places where the photographs can be stored at any given time, such as a mobile device, a cloud storage service, a memory card, a computer, or institutional servers. Security at rest describes the physical and digital security associated with data storage. While most digital cameras cannot be accessed through the internet and do not store automatically to cloud-based storage, they are also not password protected, their data is not encoded and are not usually stored as required [33]. Another disadvantage is that their information cannot be deleted remotely if the equipment is misplaced or stolen. On the contrary, a mobile device can have various layers of authentication (including passwords, fingerprints, facial recognition [2] and two-factor authentication), allow for remote deletion of content and quickly send photographs to seek advice from other experts [4, 5] or a local server through a secure network. However, it can have weaknesses that can be exploited to access local data [32], lead to unprofessional use of the images [2], and can, frequently unbeknownst to the user, be set to automatically upload to a cloud server [33].

According to GDPR, the party responsible for data storage and security must also comply with the regulations. Because most cloud storage providers have global servers and do not allow you to determine data residency, they are unsuitable for storing, even temporarily, the data in compliance with the law [19]. The optimal solution for cloud storage would be to have an institutional smartphone with cloud storage synced to the institutional servers.

 Deleting the photographs from a personal mobile device after transfer ensures the security of sensitive personal data and prevents a mixture with the owner’s data, which could cause the sensitive material to be accidentally viewed by others [7] or uploaded to a personal cloud. Even after deleting the image in a mobile device after transfer to an institutional computer or server, the device may still be storing this photograph in a folder or album dedicated to recently deleted items, and the user should remember to delete them from these folders before re-activating cloud auto-sync.

### Data transfers and security in transit

Improper transfer of data can pose as many, if not more, security risks as improper storage, and it is one of the easiest ways to intercept the data, allowing for unauthorized access. It is important to acknowledge a recurring tendency in healthcare providers to disregard security measures to prioritize convenience [5, 22]. Therefore, it is crucial to protect the transfer of images [32].

To transfer the data securely and prevent interception by third parties, the files should not be:

- Sent through unsecured, unknown, public, or dubious networks.
- Transmitted through your personal email or applications to transfer files such as WeTransfer^®^ and others, as the data will also be stored in servers of unknown location, compliance, and security [22].

Transfer the photographs to an institutional device as soon as possible [7]. If a cable is unavailable, which would be the ideal method, sending them to yourself through your institutional email using a secure network or cellular data is the safest alternative. These actions can minimize the risk of unauthorized visualization and unintended image sharing [7].

In the ideal scenario, a password-protected application where the photographs could be taken and directly stored in the institution’s database would ensure safe data transfers and storage [33], while minimising the possible third-party interceptions.

### Acceptable procedure for the capture of images using a personal mobile device

**Fig. 2:**
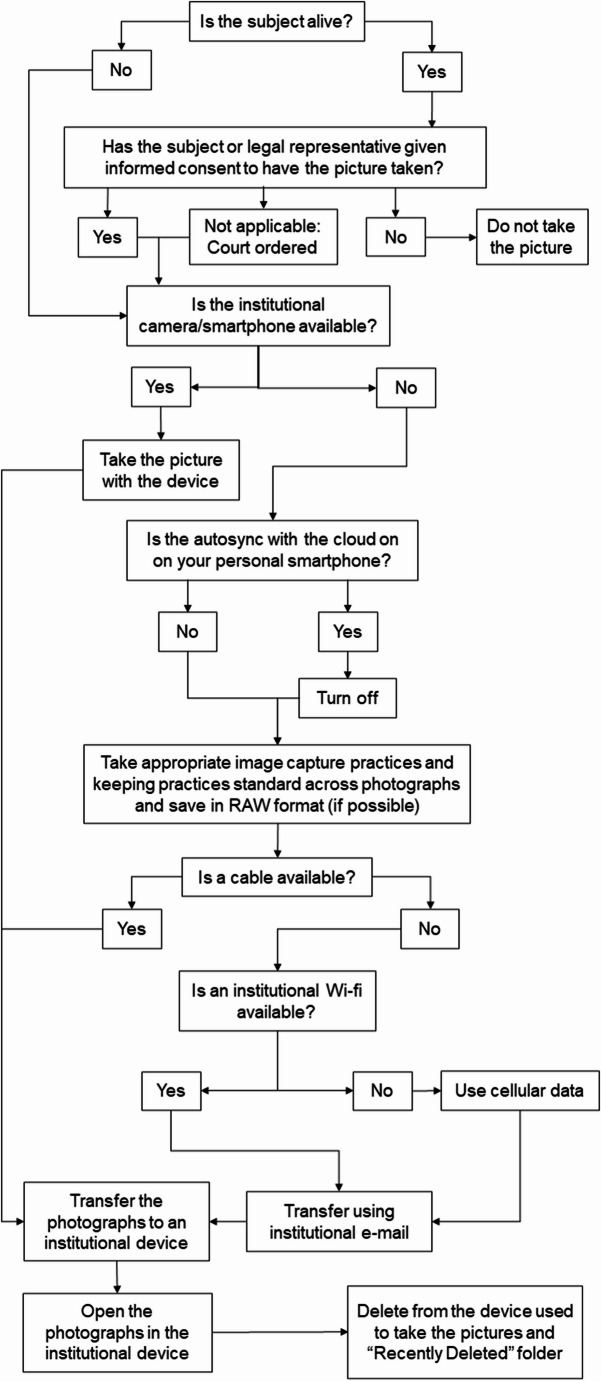
Algorithm visualization of acceptable procedure for the capture of images

A decision algorithm (see Fig. 2) was devised to summarise the essential take-home messages from the article and help implement these features of medical photography into the institutions.

The same algorithm can be used in other countries where GDPR does not apply but where there are similar health and privacy regulations. For example, the Health Insurance Portability and Accountability Act (HIPAA) is a law that was enacted in 1996, in the United States and its territories to ensure the protection and confidential handling of protected health information [34]. HIPAA establishes national standards for the protection of health information, including privacy, security, breach notification, and enforcement rules [35]. The present algorithm is also compliant with HIPAA.

## Conclusion

 With the rise of technological advancements and the emergence of advanced portable photographic devices, it is important to address their benefits and drawbacks while developing safe and acceptable practices for their future use. A basic understanding of patient privacy laws and knowing how to enhance the quality of smartphone photographs is necessary.

The authors feel it is important to note that photographs in the forensic setting should still be captured with a digital professional camera, but in light of new technological developments and in case a digital camera is not available, the forensic expert should know the correct procedure for image capture using alternative means.

## Data Availability

Data sharing not applicable to this article as no datasets were generated or analysed during the current study.

## Abbreviations

GDPR: General Data Protection Regulation
HIPAA: Health Insurance Portability and Accountability Act
